# Diagnosing heart failure with NT-proBNP point-of-care testing: lower costs and better outcomes. A decision analytic study

**DOI:** 10.3399/bjgpopen18X101596

**Published:** 2018-07-25

**Authors:** Christoffer Bugge, Erik Magnus Sether, Andreas Pahle, Sigrun Halvorsen, Ivar Sonbo Kristiansen

**Affiliations:** 1 PhD Candidate, Department of Health Management and Health Economics, University of Oslo, Oslo, Norway; 2 Senior Economist, Oslo Economics, Oslo, Norway; 3 Senior Partner, Oslo Economics, Oslo, Norway; 4 GP, Boltelokka legesenter, Oslo, Norway; 5 Professor, Department of Cardiology, Oslo University Hospital, Oslo, Norway; 6 Professor II, Cardiology Department, University of Oslo, Oslo, Norway; 7 Professor Emeritus, Department of Health Management and Health Economics, University of Oslo, Oslo, Norway; 8 Associated Partner, Oslo Economics, Oslo, Norway

**Keywords:** Heart failure, NT-proBNP, primary health care, general practice, point of care

## Abstract

**Background:**

Afflicting 1–2% of the adult population, heart failure (HF) is a condition with considerable morbidity and mortality. While echocardiography may be considered the gold standard diagnostic test, GPs have relied on symptoms and clinical findings in diagnosing the condition.

**Aim:**

The aim of this study was to estimate 1-year health outcome and costs of three diagnostic strategies: 1) history and clinical findings ('clinical diagnosis'); 2) clinical diagnosis supplemented with NTproBNP point-of-care test ('POC test') in the GP’s surgery; or (3) in hospital laboratory ('hospital test').

**Design & setting:**

A decision tree model was developed to simulate 1-year patient courses with each strategy in Norway.

**Method:**

Sensitivity and specificity of clinical diagnosis (56% and 68%), and of N-terminal pro B-type natriuretic peptide test ([NT-proBNP] 90% and 65%), were based on published literature. The probabilities of referral to hospital were based on a survey of Norwegian GPs (*n* = 103). The costs were based on various Norwegian fee schedules. Sensitivity analyses were conducted to examine the uncertainty of the results.

**Results:**

The 1-year per person societal costs were €543, €505, and €607 for clinical diagnosis, POC test, and hospital test, respectively. Even though POC entails higher laboratory costs, the total primary care costs were lower because of fewer re-visits with the GP and less use of spirometry. While 38% of patients had a delayed diagnosis with clinical diagnosis, the proportions were 22% with both POC test and hospital test. Results were most sensitive to the probability of use of spirometry.

**Conclusion:**

POC testing results in earlier diagnosis and lower costs than the other diagnostic modalities.

## How this fits in

HF is a serious condition with severely reduced life expectancy and, eventually, massive symptom burden. The clinical diagnosis can be difficult because clinical signs and symptoms are unspecific. While previous research indicates that quantification of natriuretic peptides is cost effective, this study adds that POC testing is even more cost effective when diagnosing HF.

## Introduction

HF is a clinical syndrome characterised by symptoms (such as breathlessness and fatigue) that may be accompanied by signs (such as ankle swelling, elevated jugular venous pressure, pulmonary crackles, and peripheral oedema) caused by a structural and/or functional cardiac abnormality, resulting in a reduced cardiac output and/or elevated intracardial pressure at rest or during stress.^1^ HF is not a disease in itself, but a symptom of underlying diseases such as coronary heart disease, valvular disease, or cardiomyopathy. Prognosis of HF will vary by degree and underlying cause. Although HF is a serious condition and the prognosis is usually considered to be poor, patients with mild HF may have a longevity of 10 years at the time of diagnosis. On the other hand, those with advanced disease may have life expectancy of less than 1 year. Treatment of HF involves strategies to prevent or delay the development of manifest HF or death, and to improve or reduce symptoms. Treatment plans include lifestyle changes, medication, devices, and surgical procedures.

The prevalence of HF is claimed to be 1–2% of the adult population in developed countries. While prevalence increases with age,^1^ an exact estimate is uncertain due to the lack of large population studies based on objective criteria such as echocardiography and natriuretic hormones analyses. A Danish register study found that the prevalence of HF is 0.1% in Denmark.^2^ This is probably an underestimation since it excludes patients treated in primary care setting.

Symptoms are often non-specific and may not adequately discriminate between HF and other conditions. GPs have traditionally relied on symptoms and clinical findings in diagnosing the condition, and more recently on quantification of natriuretic peptides. While echocardiography may be considered the gold standard diagnostic test,^3^ some patients are not referred for this test because of patient preferences, age, or distance to hospital.

### BNP testing

When cardiac output is lower than the body’s demand, the left ventricle becomes stretched, and the myocardial muscle cells produce increasing amounts of natriuretic peptides. Quantification of serum B-type natriuretic peptide (BNP) or NT-proBNP is therefore used as a diagnostic test of HF. Based on patients’ levels of these substances, the GP can differentiate between HF and other conditions with similar symptoms or signs.

Using test kits for various commercial analysis machine platforms, quantification of serum BNP or NT-proBNP is routinely performed in hospitals or biochemical laboratories. For example, the cobas h 232 is a handheld device designed for use in a GP’s surgery, in hospital emergency rooms, and critical care settings, or in pre-hospital situations such as in ambulances or helicopters.

With cobas h 232 GPs can read the NT-proBNP results within 8–12 minutes In comparison, testing based on blood samples sent to a biochemical laboratory normally returns results in 1–3 days. The instrument has recently undergone an independent assessment of its performance by Scandinavian Evaluation of Laboratory Equipment for Primary Health Care.^4^ The evaluation indicates good validity of cobas h 232. According to a study by Gils and others,^5^ cobas h 232 performed satisfactorily with regard to precision, user-friendliness, and lot-variation.

When GPs suspect HF, they may adopt one of three diagnostic strategies: 1) base the diagnosis on symptoms and clinical findings alone; 2) base it on symptoms and clinical findings supplemented with a natriuretic peptide test performed at a hospital-based biochemistry laboratory; or 3) base it on symptoms and clinical findings supplemented with POC natriuretic test performed in the GP’s surgery. Most GPs will also order an electrocardiography (ECG), but this examination provides information primarily on the type and cause of heart disease. Many GPs may also use spirometry for patients with dyspnoea. Still, the diagnosis of HF represents a challenge to GPs. In a systematic review of diagnostic errors in older patients, the authors conclude that up to 59% of those diagnosed with HF represented over-diagnosis. At the same time, up to 71% of those with HF could be overlooked.^6^

In 2008 Murphy and co-authors pointed out that there is a 'need for further research on the cost-effectiveness of service models for diagnosing and managing heart failure'.^7^ POC entails higher equipment costs, but may imply lower costs elsewhere in the health service or society. The results of previous studies indicate that the use of natriuretic peptides is cost effective,^8,9^ but none of the studies encompassed the use of POC in general practice when diagnosing HF.^8^ Mant *et al* state that 'future work should include evaluation of the clinical decision rule [NT-proBNP and echocardiography] … in clinical practice'.^9^ The aim of this study was to estimate the 1-year cost of the three strategies GPs may adopt to make a HF diagnosis, and the proportion of initial correct diagnoses.

## Method

### Decision model

Using TreeAge Pro Healthcare (version 2017), a decision tree model was developed to capture the 1-year diagnostic course among patients where the GP considered HF. It was assumed that the GP confirms or rejects the HF diagnosis. The result is consequently a true or false positive diagnosis, or a true or false negative diagnosis. To the extent that GPs test for natriuretic peptides, this may entail either a re-visit in person or a telephone consultation. The GP may initiate HF immediately, and/or refer the patient to a cardiologist. The model was based on numerous input parameters described in detail below.

Costs were measured as expected 1-year cost per patient from healthcare and societal perspectives. The probability of a delayed diagnosis was measured as the health outcome. A delayed diagnosis was defined as an incorrect initial diagnosis (false positive or false negative test). Results of the analyses include 1-year costs in primary care, secondary care, and patient time and travel costs, as well as health outcome (correct or incorrect diagnosis).

To gain insight into the medical management of HF among GPs, claims data were requested for the period 2007–2016 from the Norwegian Health Economics Administration (Helfo).

### Perspective

The model takes a healthcare perspective where all costs relevant to the healthcare sector were included. In addition, costs incurred from a societal perspective (patient time and travel costs) were also included. When estimating healthcare cost, the authors distinguish between costs arising in primary care and specialist care.

### Strategies

The decision model had three diagnostic strategies: history and clinical findings alone (clinical diagnosis); clinical diagnosis supplemented with NTproBNP POC test in the GP’s surgery (POC test); or in hospital laboratory (hospital test). Using NT-proBNP POC test in the GP’s surgery gives the GP the opportunity to start treatment immediately based on the NT-proBNP findings.

In each strategy, the GP may refer patients to specialist care (pulmonary or cardiological department), undertake a spirometry in the GP’s surgery, and initiate drug treatment for HF. When a test is false negative, it is assumed that symptoms will continue and that the patient will return for a re-visit.

**Figure 1.**
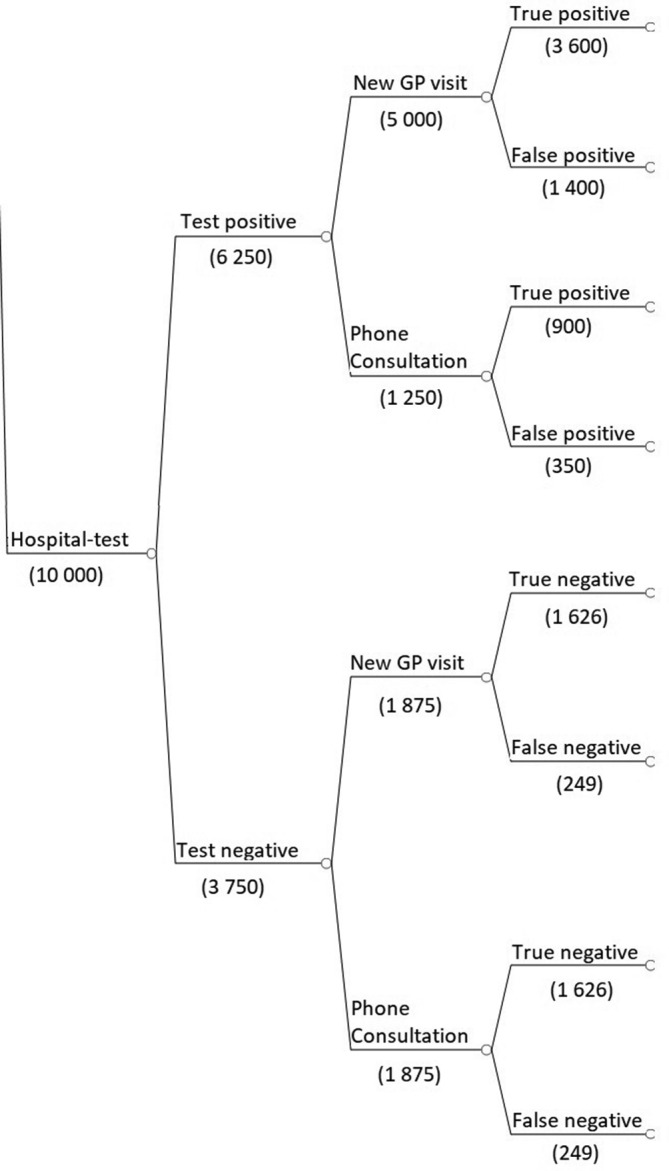
Key structure for the decision model (the number of patients is used for illustrative purposes).

**Figure 2.**
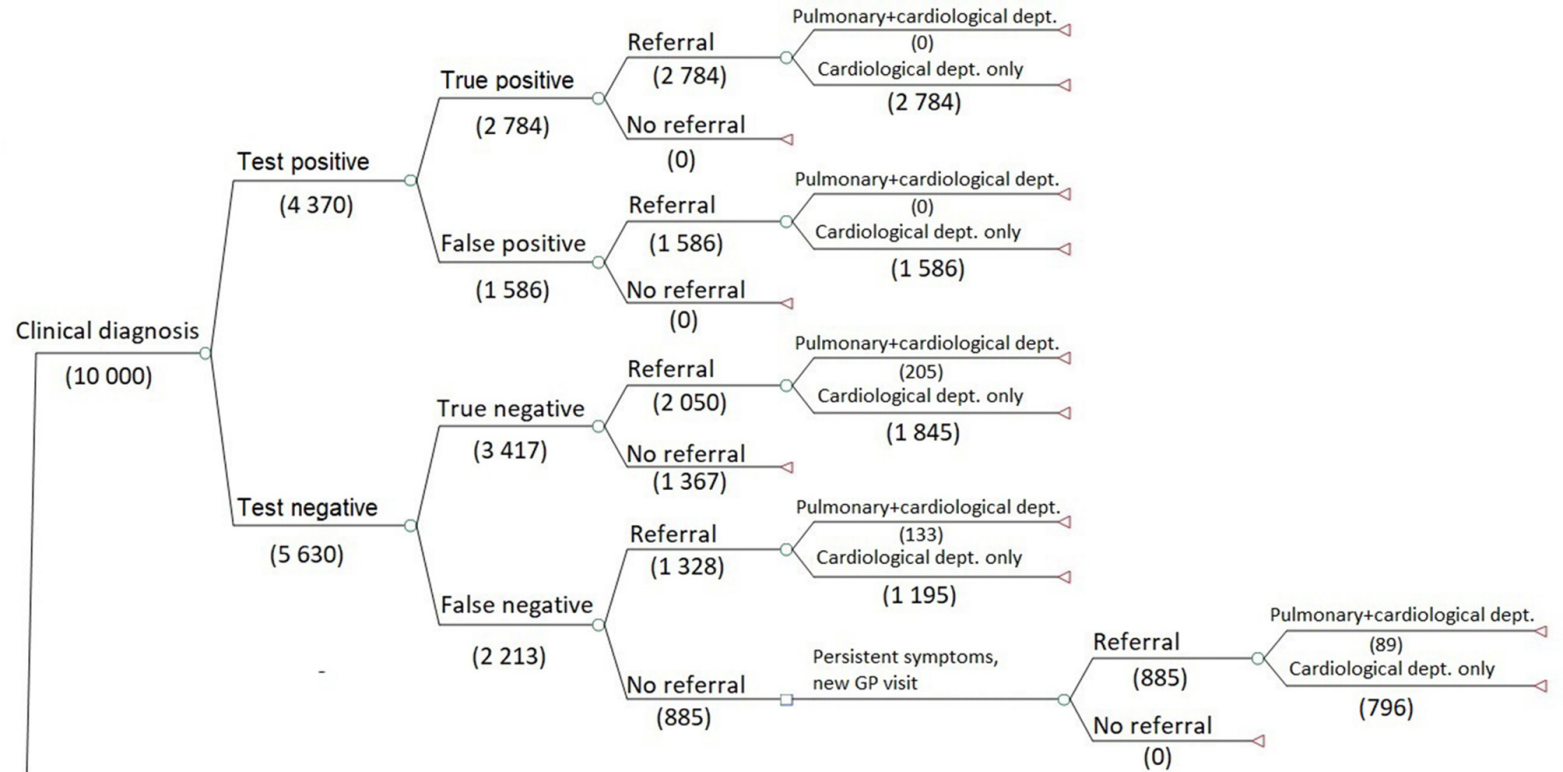
Key structure of the decision model for hospital test (the number of patients is used for illustrative purposes).

The structure of the decision tree branches is identical for the clinical diagnosis and POC test, strategies, but the probabilities of events and costs differ (see Figure 1) Branches for hospital test strategy differ as the patient can receive test results, and treatment plan by telephone or schedule a new GP visit (Figure 2). The remaining branches are identical to clinical diagnosis and POC test.

### Probabilities

The sensitivity and specificity of clinical diagnosis may vary considerably depending on factors such as the GPs’ expertise and attitude to risk. A GP may increase the sensitivity of their diagnostics at the expense of a lower specificity and vice versa. Estimates from NICE^10^ were used, while test characteristics for NT-proBNP test (90% and 65% for an age-independent cut-off value of 125 ng/L) were based on Schäfer *et al* ^11^ and Bertsch *et al* ^12^

The probabilities of referral to hospital given a test outcome were based on a convenience sample survey of Norwegian GPs (*n* = 103); see Table 1. Spirometryis reimbursed by €60 and is widely used among Norwegian GPs. To the extent GPs believe dyspnoea is caused by cardiac disease rather than pulmonary disease, POC NT-proBNP may be a substitute for spirometry. The probability of use of a spirometry versus NT-proBNP was based on the survey of Norwegian GPs, supplemented by judgment among the authors. The probability of referral to specialist care for different test outcomes, probability of referral to the pulmonary department, and probability of phone consultation for hospital test strategy were based on expert opinions by several Norwegian GPs. Sensitivity analyses were conducted to examine the uncertainty of the results.

**Table 1.** Model probabilities

Parameter	Probability (base-case value)	Lower and upper bounds	Source
**Probabilities common to all strategies**			
Prevalence of HF among patients suspected with this condition	0.5	0.45; 0.55	Expert opinion
Probability of referral to pulmonary department if negative test	0.1	0.0; 0.2	Expert opinion
Probability of referral to pulmonary department if positive test	0.0	0.0; 0.0	Expert opinion
Probability of referral to hospital if new GP visit with false negative test result	1.0	–	Expert opinion
**Clinical diagnosis strategy**			
Sensitivity	0.56	0.51; 0.61	National Clinical Guideline Centre (UK)^10^
Specificity	0.68	0.62; 0.73	National Clinical Guideline Centre (UK)^10^
Probability of referral to hospital if negative test	0.6	0.4; 0.8	Expert opinion
Probability of referral to hospital if positive test	1.0	–	Expert opinion
Probability of spirometry	0.9	0.8; 1	Expert opinion and online survey
**POC test strategy**			
Sensitivity	0.9	0.85; 0.95	Schäfer *et al* ^11^ Bertsch *et al* ^12^
Specificity	0.65	0.6; 0.7	Schäfer *et al* ^11^ Bertsch *et al* ^12^
Probability of referral to hospital if negative test	0.4	0.3; 0.5	Expert opinion
Probability of referral to hospital if positive test	1.0	–	Expert opinion
Probability of spirometry	0.2	0.1; 0.3	Expert opinion and online survey
**Hospital test strategy**			
Sensitivity	0.9	0.85; 0.95	Schäfer *et al* ^11^ Bertsch *et al^12^*
Specificity	0.65	0.6; 0.7	Schäfer *et al*,^11^ Bertsch *et al* ^12^
Probability of referral to hospital if negative test	0.4	0.3; 0.5	Expert opinion
Probability of referral to hospital if positive test	1.0	–	Expert opinion
Probability of phone consultation if positive test	0.2	0.1; 0.3	Estimate based on online survey
Probability of spirometry	0.8	0.7; 0.9	Expert opinion and online survey

HF = heart failure.

### Costs

The Norwegian fee schedule for GPs^13^ and diagnosis related groups (DRG) price list for estimates of unit costs in health care were used (Table 2). For the POC test strategy, test costs and the cost of time required to conduct the test were added. Estimates for fixed costs (depreciation and maintenance of the device) were based on the current market price of cobas 232 (NOK 15 374, excluding VAT), an expected lifetime of 8 years, and the assumption of 140 tests per machine per year. Estimates for operating costs (test-kit and share of quality control costs) were delivered by Roche Diagnostics Norway.

**Table 2.** Model costs (€1.00 [2017 Euro] = NOK 9.00)

Parameter	Base-case value, €	Lower and upper bounds, €	Calculation method or source
**Healthcare costs**			
GP visit	32	22; 41	Fee schedule
Spirometry	60	54; 66	Fee schedule
Other test in GP surgery (ASAT, ALAT, potassium, chloride, sodium, ECG, cholesterol [total, HDL, and LDL], creatinine)	23	16; 30	Fee schedule
1-year use of diuretics, beta-blockers, and aldosterone antagonist	89	62; 115	Norwegian Pharmaceutical Product Compendium
Outpatient visit	282	198; 366	Fee schedule and DRG weights
Investment and maintenance of POC machine	1	1; 2	Estimation based on depreciation over 8 years, 140 tests per machine per year
Test-kit POC	28	20; 36	Expected price and share of costs quality control
GP telephone consultation	7	5; 9	Fee schedule
Sending test to laboratory	6	4; 7	Fee schedule
Laboratory test	6	4; 8	Fee schedule
**Patient time and travel costs**			
Patient time costs, GP visit (2 hours)	45	32; 59	Net annual earnings
Patient time costs, specialist visit (3 hours)	68	47; 88	Net annual earnings
Patient travel costs, GP visit	22	16; 29	Moger *et al* ^15^
Patient travel costs, specialist visit	34	24; 44	Moger *et al* ^15^
Additional patient time costs if POC testing (20 minutes)	8	5; 10	Net annual earnings
Patient time cost, telephone consultation	4	3; 5	Net annual earnings

ALAT = alanine aminotransferase. ASAT = aspartate aminotransferase. DRG = diagnosis related groups. ECG = electrocardiography. HDL = high density lipoprotein. LDL= low density protein. NOK = Norwegian Krone. POC = point of care.

Since most HF patients in Norway are aged >60 years,^14^ time costs were assumed to be lost leisure, using net annual earnings to estimate costs of lost leisure. Patients’ travel costs were based on Moger and Kristiansen.^15^ Discounting was not performed, as the time perspective was 1 year. All costs were presented in 2017 Euros (Table 2).

### Sensitivity analysis

Probabilistic sensitivity analyses were performed using Monte Carlo simulation (5000 simulations), using TreeAge PRo Healthcare (version 2017). The results were presented as cost-effectiveness acceptability curves and scatter plots. Estimates of uncertainty were based on expert judgment.

## Results

The expected 1-year healthcare cost of the clinical diagnosis strategy was €379, €397 for hospital test, and €344 for POC test (Table 3). Expected 1-year societal cost for the clinical diagnosis was €543, €607 for hospital test, and €505 for POC test. POC test had lowest costs in both primary (€114) and secondary care (€231), as well as time and travel costs (€158), almost equal to clinical diagnosis. While the hospital test strategy had highest costs in primary care (€161), the clinical diagnosis strategy implied highest costs in secondary care (€254).

**Table 3.** Expected 1-year costs for different strategies (€1.00 [2017 Euro] = NOK 9.00)

Strategy	Healthcare cost, € (95% CI)			Patient time and travel costs, € (95% CI)	Societal cost, € (95% CI)	Proportion of initial incorrect diagnosis, % (95% CI)
	Primary care	Specialist care	Total			
Clinical diagnosis	130(68 to 281)	254 (124 to 430)	379(226 to 601)	160 (116 to 214)	543 (378 to 767)	38.0 (31.0 to 45.0)
Hospital test	161(133 to 194)	237 (121 to 391)	397(276 to 554)	207 (153 to 275)	607 (469 to 780)	22.0 (16.0 to 31.0)
POC test	114(91 to 141)	231 (116 to 383)	344(225 to 502)	158 (116 to 210)	505 (375 to 674)	22.0 (16.0 to 31.0)

CI = confidence interval. NOK = Norwegian Krone. POC = point of care.

The proportion of initial incorrect diagnosis was 38% for clinical diagnosis, and 22% for both hospital test and POC test.

National reimbursement claims data were used to validate the prevalence of HF among GP patients, but the data would indicate a prevalence that is not correct. The annual number of patients with a HF diagnosis varied between 4856 and 5408 during the period 2007–2016.

**Figure 3.**
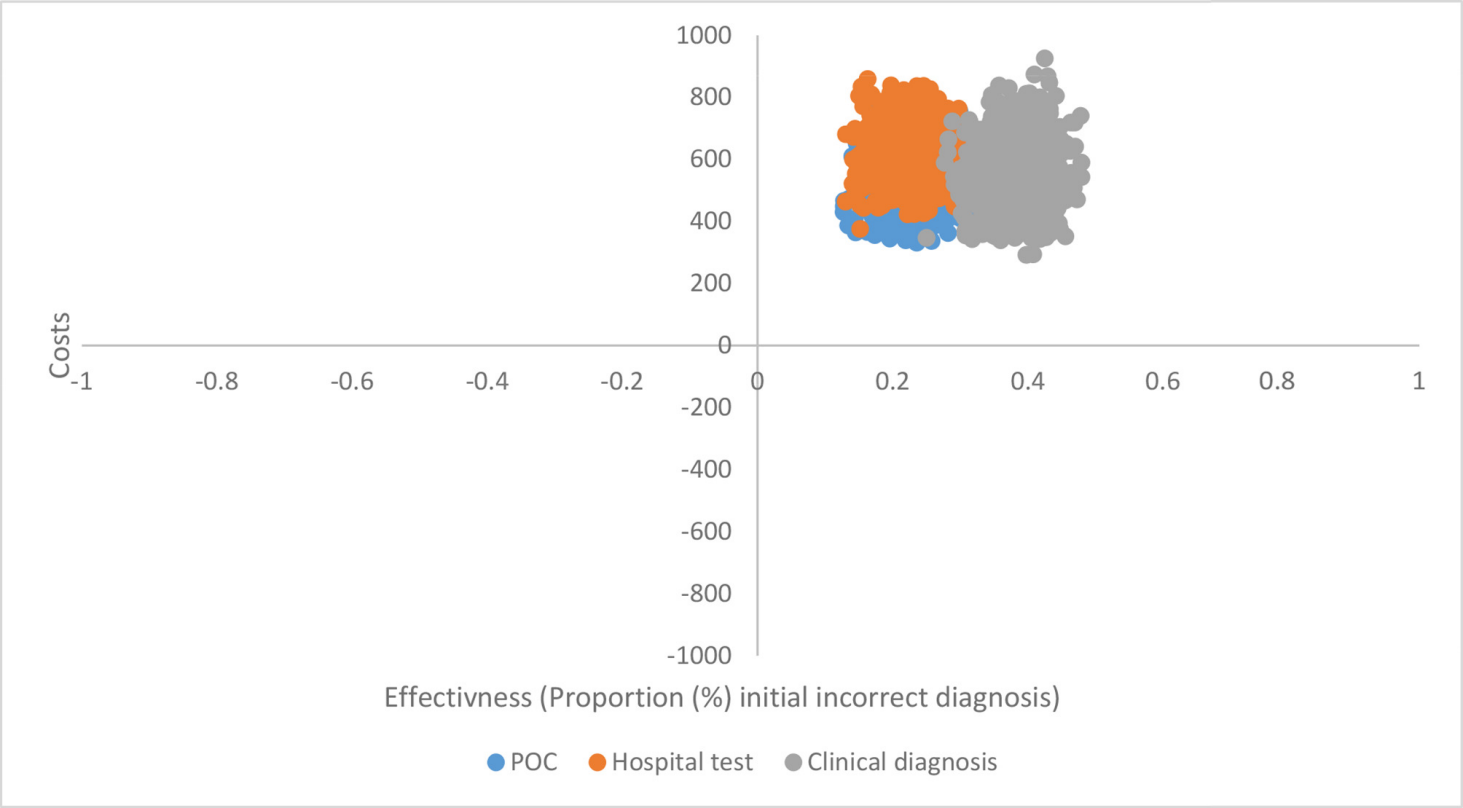
Cost-effectiveness scatterplot (societal cost in 2017 Euros).

Sensitivity analysis support findings that the POC strategy entails lower costs and better health outcome in terms of earlier diagnosis, as the cost-effectiveness scatterplot (Figure 3) has limited overlap between the three strategies. Additional one-way sensitivity analysis showed that POC was the optimal strategy if the probability of spirometry is ≤81%.

## Discussion

### Summary

The results of this study indicate that POC testing results in earlier diagnosis and lower costs than the other diagnostic modalities. This conclusion, however, should be seen against the limitations of the study.

### Strengths and limitations

The model is necessarily based on a number of uncertain assumptions. This is especially true for the probability that the GP uses spirometry, and the prevalence of HF among patients suspected with the disease. It is also uncertain whether NT-proBNP will, in reality, replace spirometry. Furthermore, follow-up routine practice differs between GPs, which makes treatment pathways difficult to model. The authors had hoped that claims data would assist in describing treatment pathways but, unfortunately, the low number of patients registered with HF indicates incomplete registration. In addition, the model has a 1-year horizon only, and some costs are therefore not included. This applies, for example, to costs associated with diagnostic errors. When calculating costs, fee schedules were used which may not reflect the actual societal cost.

Although health outcome only was captured as proportion of correct initial diagnosis, there is little reason to expect that other measures of health outcome would change the conclusion that POC testing entails lower costs and equal or better outcome than the other strategies. Sensitivity analyses support findings, and changes in key parameters have limited impact on the results. To ensure that NT-proBNP replaces spirometry, the government can impose restrictions on spirometry refunds.

Unfortunately, the claims data indicate that the ICPC diagnosis is so rarely used that data cannot be used to describe the medical management or the prevalence of HF. GPs are likely to report the underlying disease (for example, coronary disease), but not HF.

### Comparison with existing literature

In a systematic review, Athanasakis *et al* ^8^ demonstrate that testing for natriuretic peptides is cost-effective in the majority of studies available in the literature. The authors point out that relevant studies indicate that testing for natriuretic peptides reduces total cost of HF treatment per patient by 7–34%, and that savings are related to reduction in admissions and readmission rates as well as hospitalisation days. The present study adds to existing evidence in that POC testing of natriuretic peptides is even more cost-effective than testing in a laboratory when diagnosing HF in general practice, mainly since spirometry is less frequently used.

### Implications for practice

The main reasons that POC testing is used only to a limited extent in Norway likely lies in GPs’ lack of knowledge about the test, and the lack of reimbursement for the test. Introducing reimbursement for the test will increase GPs incentives to use it, which again can lead to socioeconomic savings in terms of lower costs and better health outcomes. Government payments will increase as a result of the new technology, while payments related to spirometry will be reduced. It should be noted that the impact of financial incentives on GP behaviour is controversial.^16^

In conclusion, POC NT-proBNP test seems to entail lower healthcare costs in both primary and secondary care, reduced travel and time costs for patients, and better outcome (earlier diagnosis).

The study was presented orally at Society of Medical Decision Making‘s biennial meeting London 2016 and WONCA, Copenhagen 2016.
